# Cause and effect of microenvironmental acidosis on bone metastases

**DOI:** 10.1007/s10555-019-09790-9

**Published:** 2019-03-02

**Authors:** Sofia Avnet, Gemma Di Pompo, Silvia Lemma, Nicola Baldini

**Affiliations:** 1 Orthopaedic Pathophysiology and Regenerative Unit, IRCCS Istituto Ortopedico Rizzoli, Bologna, Italy; 2 Department of Biomedical and Neuromotor Sciences, University of Bologna, 40123 Bologna, Italy

**Keywords:** Bone metastases, Acidosis, Osteoclast, Tumor microenvironment, Bone resorption, Pain

## Abstract

Skeletal involvement is a frequent and troublesome complication in advanced cancers. In the process of tumor cells homing to the skeleton to form bone metastases (BM), different mechanisms allow tumor cells to interact with cells of the bone microenvironment and seed in the bone tissue. Among these, tumor acidosis has been directly associated with tumor invasion and aggressiveness in several types of cancer although it has been less explored in the context of BM. In bone, the association of local acidosis and cancer invasiveness is even more important for tumor expansion since the extracellular matrix is formed by both organic and hard inorganic matrices and bone cells are used to sense protons and adapt or react to a low pH to maintain tissue homeostasis. In the BM microenvironment, increased concentration of protons may derive not only from glycolytic tumor cells but also from tumor-induced osteoclasts, the bone-resorbing cells, and may influence the progression or symptoms of BM in many different ways, by directly enhancing cancer cell motility and aggressiveness, or by modulating the functions of bone cells *versus* a pro-tumorigenic phenotype, or by inducing bone pain. In this review, we will describe and discuss the cause of acidosis in BM, its role in BM microenvironment, and which are the final effectors that may be targeted to treat metastatic patients.

## Introduction

Skeletal involvement is a frequent and troublesome complication affecting many patients with advanced cancer. Bone is the third most common metastatic site after the lung and the liver [1]. Up to 85% of patients dying from breast, prostate, or lung cancer reveal bone involvement at autopsy [2]. Once tumor cells are housed in the skeleton and form bone metastasis (BM), the disease is usually incurable and treatment with current modalities is only palliative and often inadequate and causes uncomfortable side effects.

Invasion of bone compartment by cancer cells results from different mechanisms that include the homing of malignant cells to bone marrow niches and the acquisition of osteomimicry [3], an osteomimetic cell phenotype that allows tumor cells to interact with cells of bone microenvironment and seed in the bone tissue. Once tumor cells home in the bone tissue, they affect the balance between the activities of the bone forming cell, the osteoblast, and the activities of the bone-resorbing cell, the osteoclast, thereby causing the formation of predominantly osteogenic or osteolytic lesions [4]. Typically, osteolytic metastases are clinically more relevant than osteoblastic metastases, which have a slower course and are not associated with bone frailty and pathological fractures. In addition to osteoblasts and osteoclasts, other stromal cells may contribute to BM progression, including mesenchymal stromal cells (MSC) [5] that are the osteoblast precursors, osteocytes [6, 7], immune cells, and nerves that are responsible for cancer-induced bone pain (CIBP) [8]. As a part of the tumor microenvironment (TME), mineralized bone matrix is another critical player to set the scene for the development of BM since its degradation releases mitogenic growth factors and calcium ions. Furthermore, uncontrolled proliferative cancer cells are highly glycolytic and thus secrete substantial amounts of proton/lactate into the extracellular environment, a phenomenon known as the Warburg effect [9–12]. As in other solid tumors, interstitial acidosis in BM can also be derived from hypoxia (low oxygen tension). According to Darwinian evolutionary theory, cancer cell populations rapidly converge to the fittest phenotype given a stable environment. Thus, like for other tumors, extracellular acidosis BM is a regional variation created by tumor cells and to which tumor cells have to adapt and take advantage to become even more aggressive [13]. In fact, tumor acidosis has been directly associated with tumor invasion and aggressiveness in several types of cancer [11] (and other contributions to the volume). In bone tissue, the association of local acidosis and cancer invasiveness is even more important for tumor expansion since the extracellular matrix (ECM) is formed by both organic and hard inorganic matrices. However, in the context of BM, the increased concentration of protons in the TME may derive not only from glycolytic tumor and inflammatory cells [14, 15], as found in other solid tumors, but also from tumor-induced osteoclasts that dissolve the mineralized matrix [16]. The induction of abnormal extracellular acidification in the TME may influence the progression of BM in different ways, by directly enhancing cancer cell motility and aggressiveness, by modulating the functions of bone cells *versus* pro-tumorigenic effects, or by inducing bone pain.

In this review, we will describe and discuss the cause of acidosis in BM, how it is detected within the BM and which are the final effectors that might be targeted to treat bone metastatic patients in the future.

## The formation of acid TME in bone metastasis

The abnormal pH gradient in the TME is finely tuned by a number of ion/proton pumps that are expressed both in tumor cells and in tumor-associated normal cells. Among these, the vacuolar H^+^-ATPase (V-ATPase) has been identified as the most important for BM progression, since it is expressed both in cancer cells and osteoclasts.

**Fig. 1:**
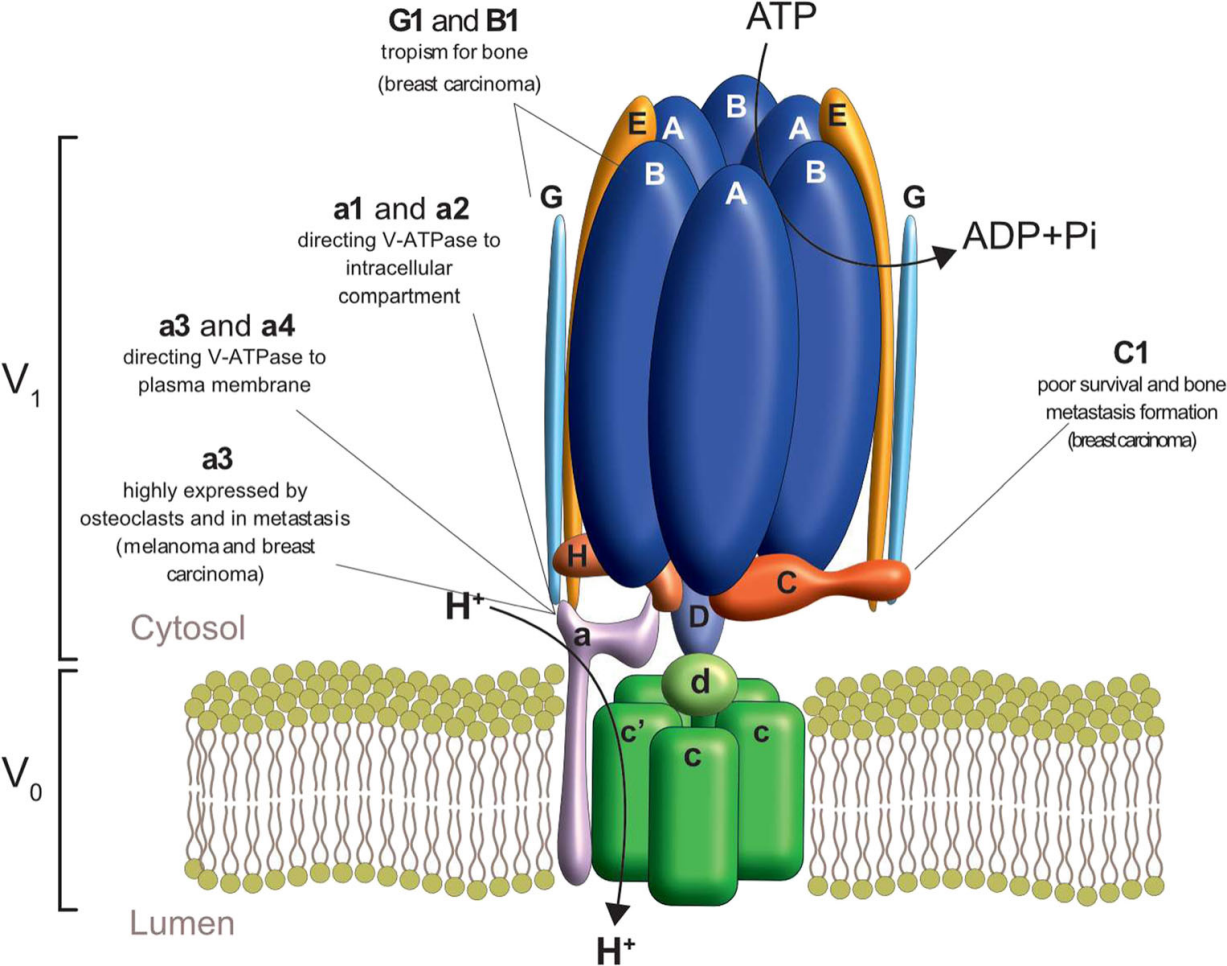
V-ATPase subunits in BM. The V-ATPase complex is formed by a peripheral domain (V_1_) responsible for ATP hydrolysis, and an integral domain (V_0_) that is involved in the translocation of protons across the cell membrane. The V_1_ domain is formed by a hexameric core of A-B subunits that participate to ATP binding and hydrolysis, and other seven ancillary proteins responsible for the rotation of the central core. The V_0_ domain includes a ring of proteolipid subunits inserted in the lipid bilayer. The role of V-ATPAse subunits that are relevant in BM is highlighted

V-ATPase is a family of ATP-powered proton pumps that are mainly located on the lysosomal membrane and acidify the intralysosomal space. In highly acidifying cells, V-ATPase can be also found on the cytoplasmic membrane to pump protons directly outside the cell, as in osteoclasts and this, in turn, activates acid proteases and degrades the ECM [17, 18]. V-ATPase is formed by an ATP-hydrolytic domain (V1) and a proton-translocation domain (V0) (Fig. 1). The V1 domain includes eight subunits (A-H). The membrane-embedded V0 domain has five subunits (a, c, c″, d, e) [19]. V-ATPase activity requires the tight association of all the components of the complex, which is ensured by the C ring [20–22]. The targeting of V-ATPase to different cellular membranes is controlled by isoforms of subunit a, with a1 and a2 isoforms directing V-ATPase primarily to intracellular compartments, and a3 and a4 directing the proton pump to the plasma membrane [23, 24]. V-ATPase has also several other cellular functions, like mediating Notch receptors and Wnt or mTORC signaling pathways [25].

In addition to V-ATPase, other proton extruders have been associated with cancer [2], like Na^+^/H^+^ exchanger (NHE), monocarboxylate transporters (MCT), and carbonic anhydrase 9 (CAIX) [11]. Although in the context of the BM microenvironment these proton extruders have been extensively investigated in osteoclasts, their role in cancer cells that develop BM remains still unclear.

### Extracellular acidification by cancer cells

The a3 subunit of V-ATPase has been correlated to the metastatic potential of melanoma and breast carcinoma cells [26–28]. Also, the Atp6v1c1, an isoform of the C subunit, is highly overexpressed or amplified in 34% of human breast cancer cases and is associated with poor survival, breast cancer growth, and BM formation [29]. The knockdown of the respective gene reduces the local acidification by tumor cells and osteoclast formation thereby affecting metastasis occurrence *in vivo* [29]. Other subunit isoforms of V-ATPase have been associated with a more aggressive cancer phenotype or with a specific tropism for bone: in a subclone of MDA-MB-231 breast cancer cells that are more keen to metastasize to bone with respect to the parental cell line, we observed a higher level of expression of the V_1_B_1_ and V_1_G_1_ isoforms, both under normoxia and hypoxia [30]. Regarding the other proton/ion transporters, not much has been described. Among the few examples, it has been reported that MCT4 is more highly expressed in metastases to bone relative to other metastatic sites, like brain, lung, and liver [31], and that MCT4 expression in tumor cells allows the metabolic coupling of tumor cells and osteoclasts, thereby inducing a higher osteolytic activity in BM from breast carcinoma [32].

### Extracellular acidification by osteoclasts

Osteoclasts are very specialized cells that can resorb large amounts of mineral and organic bone matrix [33, 34]. As giant multinucleated, non-proliferative polykaryons, osteoclasts form through fusion of mononuclear precursor cells [35] in response to two major cytokines: the macrophage colony-stimulating factor (M-CSF) and the receptor activator of nuclear factor-kappa B (NF-κB) ligand (RANKL) [33, 36, 37]. The process of bone degradation in osteoclasts deeply relies on the secretion of H^+^ through the fusion of vesicles to a specific late endosome-like domain of the plasma membrane facing the bone, the so-called ruffled border. The formation of the ruffled border occurs as a consequence of osteoclast polarization through drastic changes in the organization of the cytoskeleton and vesicle transport, which is followed by hydrochloric acid secretion [38]. In this process, the endocytotic pathway is re-oriented from the basolateral membrane and the biosynthetic pathway of lysosomal enzymes from the Golgi toward the bone surface [38]. Bone resorption therefore depends on the expression and activity of H^+^-secreting proteins at the ruffled border, which is primarily the V-ATPase. The demonstration of the formation of an acidic area adjacent to the polarized osteoclasts was first made through the use of the fluorescent probe acridine orange [39]. Later, it was shown—using pH microelectrodes—that osteoclasts can acidify the contact zone with a culture dish to less than pH 3 within a few minutes [40]. In osteoclasts, the different roles and actions of V-ATPase have been documented and characterized already from 1989 [41]. Among the different subunits, despite being widely expressed in most human tissues, a3 is especially crucial for bone resorption [42]. Nonetheless, a3 expression is induced during osteoclast formation and is 100-fold higher in osteoclasts compared to other cell types [43]. However, additional mechanisms have been implicated in the acidification of the resorption lacuna: osteoclast acidification occurs under continuous flow conditions and exhibits progressive intracellular acidification, accompanied by spontaneous rhythmic oscillations of intracellular pH. The regulation of intracellular pH is related to the translocation of several ions across the plasma membrane by specific proteins [44], like Cl^−^ secretion by chloride channel 7 (ClC-7) [44, 45]. In this process, intracellular pH is also maintained by H^+^ conductance and the activity of the NHE10 isoform [44, 46], or by the base-transporters NBCn1 (Na^+^-HCO3^−^ co-transporter) [47] (see Bødkjer, this volume), and AE2 (Cl^−^/HCO3^−^ anion exchanger) [48]. Notably, although NHE1 is involved in osteoclast formation and function, it is not involved in lacunae acidification [49, 50].

### Extracellular acidification, an energy-consuming activity

Very active cells like osteoclasts require an extremely high metabolic activity. To support the energy-consuming process of proton extrusion, *de novo* synthesis and compartmentalization of glycolytic enzymes, like glyceraldehyde 3-phosphate dehydrogenase (GAPDH) and pyruvate kinase muscle isozyme 2 (PKM2), are required close to the bone interface and the sealing zone during bone resorption for the rapid generation of ATP and the efficient functioning of V-ATPases [51, 52]. However, the maintenance of the basal cellular activities of such large, complex, and multinucleated cells also demand for *de novo* mitochondrial biogenesis [51]. Thus, in the context of the BM microenvironment, it is reasonable to speculate that hypoxic and glycolytic metastatic cells that colonize the bone will stimulate osteolysis, not only by the activation of pro-osteoclastogenic molecular pathways but also through the fueling and maintenance of osteoclast mitochondrial functions. Indeed, taking in consideration the metabolic nature of osteoclasts, from one side the extracellular accumulation of metabolites, such as lactate and serine, might stimulate osteoclastogenesis or might fuel oxidative metabolism [32, 53–55], whereas, as demonstrated in neurons, the acidification of the TME might contribute to the homeostatic program that regulates mitochondrial dynamics and cristae architecture, to reconfigure mitochondrial efficiency and maintain mitochondrial function (see Sonveaux, this volume) and cell survival of osteoclasts [12, 56], thereby inducing long-lasting bone resorption.

## The acidifying invadosomes in bone metastases

**Fig. 2:**
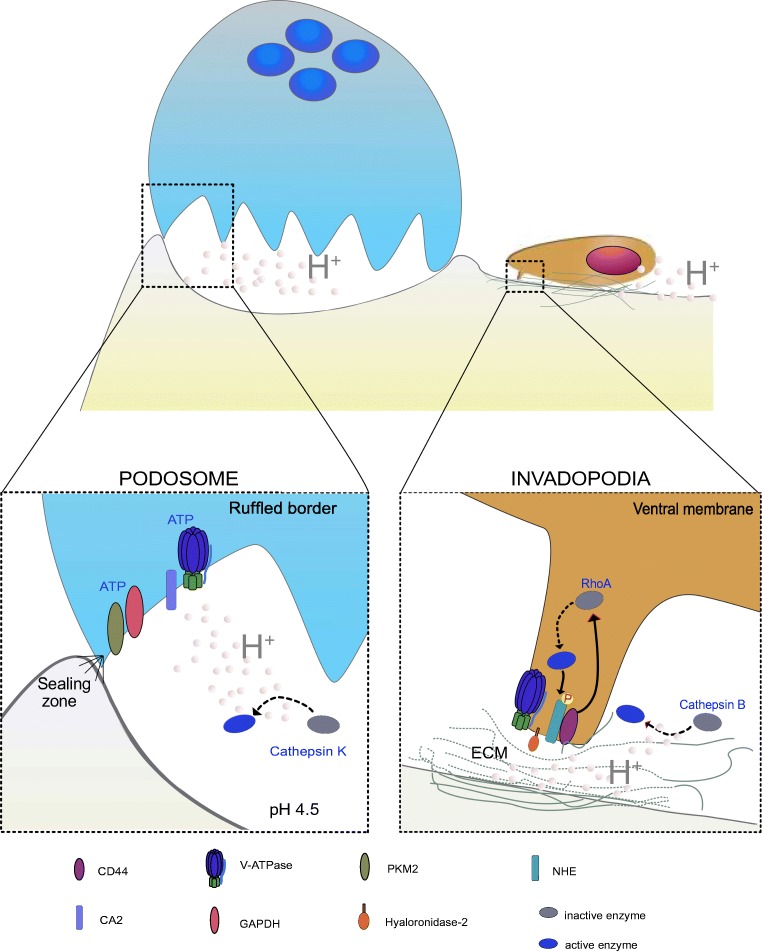
Invadosome in BM. Podosome is a characteristic of normal cells such as osteoclasts, whereas invadopodia develop at the leading edge of tumor cells. The organization of highly specialized podosome-based ring-like structure forms the sealing zone to concentrate protons into an isolated compartment between the osteoclast and the matrix to degrade bone. CA2, V-ATPase, GAPDH, and PKM2 localize in the ruffled border close to the sealing zone to allow the rapid generation and secretion of protons into the Howship’s lacunae. In invadopodia, the external portion of CD44 binds to ECM components, whereas the intracellular domain of CD44 interacts with specific signaling molecules which activate the RhoA enzyme that in turn phosphorylates NHE1 and induce extracellular acidification and activation of cathepsin B and hyluronidase-2 needed for ECM degradation and tumor cell invasion

Invadosomes are key functional adhesive structures similar to focal adhesions that establish close contact with and degrade the ECM. Among the invadosomes, podosomes form in normal cells that cross or remodel tissue barriers, whereas invadopodia form in cancerous or transformed cells (Fig. 2). Podosomes and invadopodia share molecular composition, participation in cell-matrix adhesion, and promotion of ECM degradation, but differ for structure organization, size, number, and half-life. Podosomes are non-protrusive, highly dynamic, small structures (1 μm × 0.4 μm) of the invading membrane that extend into the intracellular space and are constituted by a central F-actin-rich core surrounded by a ring of integrins [57] and associated proteins that pave the attachment to the extracellular matrix [58]. Podosomes develop in osteoclasts [59], macrophages [60], dendritic cells [61], endothelial cells [62], and vascular smooth muscle cells [63], and may promote motile behaviors observed in cancer cells. Invadopodia are a larger, more persistent, and more protrusive structure (8 μm × 5 μm) extending in the ECM relative to podosomes. The core of invadopodia is made of F-actin filaments but lacks the rings of integrins. Podosomes are found in high quantities, often more than 100 units per cell, whereas there are only a few invadopodia per cell [64, 65].

### Invadopodia in tumor cells

Invadopodia develop on the ventral membrane of invading cells (Fig. 2) and are primarily involved in the focal degradation of ECM through the spatial and temporal release of proteases and protons at the leading edge of invading cells [50, 64]. The directed movement of an invasive cell requires dynamic remodeling of the cytoskeleton and redistribution of activated V-ATPases and NHE1 to the tip of the invadopodia compartment [66]. In the same compartment, V-ATPase containing vesicles may co-localize also with F-actin, near to the leading edge of the migrating tumor cells, like in prostate cancer [67]. Acidic microenvironments may be created also by the interaction of CD44 and NHE1 into caveolin-1-containing lipid rafts, like it has been demonstrated in breast cancer cells [68]. In more detail, the binding of hyaluronan, a major component of ECM, to CD44 activates Na^+^-H^+^ exchange activity, which in turn promotes both intracellular pH perturbation and creates an acidic extracellular matrix environment. In more details, when CD44 binds to hyaluronan, the intracellular compartment of CD44 interacts with signaling molecules which stimulates RhoA-mediated Rho kinase activity that, in turn, increases the serine/threonine phosphorylation of both the adaptor protein Gab-1 [69] and NHE [68]. Phosphorylated Gab-1, in turn, promotes phosphatidylinositol 3-kinase recruitment to CD44, whereas NHE1 phosphorylation leads to hyaluronan catabolism [68], and enhanced lysosomal trafficking [70]. Lysosomes are the major storehouse of cellular proteases and their increased turnover at the cell periphery leads to the secretion of lysosomal proteases, including cysteine proteinase that are activated by low pH and that can be therefore directly activated by NHE activity (e.g., cathepsin B) [68]. NHE1 is also involved in a mechanism of acidification of the peri-invadopodial space, which *per se* regulates invadopodia formation and maturation [66, 71]. In the context of BM, Na^+^/H^+^ exchanger regulatory factor (NHERF1) also recruits membrane and cytoplasmic and cytoskeletal signaling proteins into functional complexes to regulate breast cancer organotropism and BM invasiveness to the bone. Indeed, the PDZ2 domain of NHERF1 fosters the formation of visceral metastases *via* increased invadopodia-dependent invasion and anchorage-independent growth, as well as by inhibition of apoptosis, whereas the PDZ1 domain fosters the formation of BM through the stimulation of podosome nucleation, motility, neoangiogenesis, vasculogenic mimicry, and osteoclastogenesis [72].

### Podosomes in osteoclasts

Osteoclast function primarily depends on their ability to extrude protons and proteases through the ruffled border into a small cavity, named the Howship’s lacuna, to digest the underlying bone. The Howship’s lacuna is a specialized compartment that is tightly secluded and delimited by an adhesive structure, the sealing zone, to allow the formation of a very acid pH with a high concentration of bone digesting enzymes. The sealing zone forms between the osteoclast ventral membrane and the bone matrix through the fusion of podosomes. Podosomes arrange in different patterns according to the osteoclast function, i.e., for migrating or resorbing bone. To resorb bone, the osteoclast polarizes and the podosome-containing sealing zone forms a ring-like structure. Each podosome has an F-actin-enriched central core surrounded by a ring that is formed by CD44, integrins, and integrin-associated proteins which mediate the adhesion to the ECM. Protons and acid proteases released through the ruffled border produced a pH drop to 4.5–4.8 that allows the dissolution of the mineralized inorganic bone matrix, hydroxyapatite, and the activation of acid proteases, mainly cathepsin K, to degrade the organic bone matrix [39, 73, 74]. pH regulators like CA2 and V-ATPase localize in close proximity to podosomes at the ruffled border [75]. In particular, the a3 isoform of V-ATPase that resides in lysosomes is re-localized to the ruffled border upon osteoclast activation [76].

## Bone cells are proton-sensing machines

Bone cells sense protons and adapt or react to a low pH, both under physiological conditions or under altered conditions, like inflammation, to maintain tissue homeostasis (Table 1). Also in the BM microenvironment, bone cells can perceive tumor acidosis and react to such stress signals, possibly fostering tumor progression.

**Table 1:** Extracellular acidic pH effects on bone cells and the role of proton-sensing GPCRs and ion channels

Receptors	Bone cell types	Acidification conditions	Signalings and functions	Acidification-induced actions	Ref.
OGR1	Osteoblasts	pH 7.6–6.3	Gq/11/PLC/Ca^2+^	COX-2 expression, PGE2 production	[77]
	Osteoclasts	pH 7.6–7.0	Ca^2+^/calcineurin/NFATc1	Osteoclastogenesis and bone resorption	[78]
	Osteoclasts	pH 7.6, pH 7.0	Protein kinase	Osteoclast survival	[79]
GPR4	Osteoblasts	pH 8–6	cAMP accumulation	RANKL expression	[80]
TDAG8	Osteoblasts	pH 7.4–6.88	cAMP accumulation	N/A	[80]
	Osteoclasts	pH 7.4	cAMP accumulation	Inhibition of bone resorption	[81]
ASIC1-3	Osteoblasts	pH < 7	Proton channel	N/A	[82]
	Osteoclasts	pH < 7	Proton channel	Possible role in monocyte differentiation and osteoclast survival	[82]
TRPV1	Osteoblasts	pH 5–4	Proton channel	Osteoblast differentiation and function	[83]
TRPV1, 2, 4, 5	Osteoclasts	pH 5–4	Proton channel	Osteoclast differentiation and function	[83, 84]

Both osteoblasts and osteoclasts express proton sensors that belong to the family of acid-sensing ion channels (ASIC) and transient receptor potential vanilloid (TRPV) ion channels that are typically expressed by sensory neurons, including ASIC 1–3 [82], TRPV1 [85], and TRPV4 [86]. In more detail, several TRPV channels are involved in osteoclast differentiation, including TRPV1 and TRPV2 [83, 84], as well as TRPV4 channels, which appear to be, at least in part, implicated in acidosis-dependent large cell formation. Additional components of the acid-sensing machinery in osteoclasts are ASIC1, ASIC2, and ASIC3, which are expressed in both monocytes and differentiated osteoclasts, with ASIC2 being the most abundant, whereas ASIC4 mRNA is virtually absent in both cell types [82]. Metabotropic proton-sensing G protein-coupled receptors (GPCR) have also been recently identified as proton-sensing machinery in bone [80, 87, 88]. Of note, while ASIC and TRPV ion channels are activated by moderate decreases in extracellular pH (pH 7–4) [89] and by severe acidosis (pH 5–4) [90], respectively, GPCR can sense more physiological or weakly acid pH (pH 8–6) [90]. The family of GPCR includes the ovarian cancer G protein-coupled receptor 1 (OGR1, also known as GPR68), the G protein-coupled receptor 4 (GPR4) [88], the T cell death-associated G protein 8 (TDAG8, also known as GPR65) [91], and the G2 accumulation protein (G2A, also known as GPR132) [92]. These receptors are coupled either to phosphoinositide metabolism and increased intracellular calcium (OGR1 and G2A) [88, 92] or to alteration in adenylate cyclase activity (GPR4 and TDAG8) [88, 91] that are both strongly involved in the regulation of bone cell functions in response to acidosis.

Among GPCRs, OGR1 plays a primary role as a proton sensor in osteoblasts. Ludwig et al. [88] first reported the expression of OGR1 protein in active osteoblasts and lining cells on the bone surface. Subsequently, other authors confirmed mRNA expression of OGR1 in both human [77] and mouse [93] osteoblasts. Besides being the main pH sensor in osteoblasts, OGR1 seems to play a direct role in the response of osteoclasts to an acidic microenvironment. Its levels appear to be increased in osteoclasts under RANKL and CSF-1 treatments [94], and depletion of OGR1 results in decreasing cell survival [79], providing evidence of the pivotal role of this receptor in the biology of bone-resorbing cells. Further evidence for a positive role of OGR1 in osteoclastogenesis was demonstrated in OGR1 knockout mice: osteoclast survival was correlated to pH, even if no overall skeletal abnormalities were observed [95]. Finally, osteoblasts also express the GPR4 receptor [89], which mediates RANKL expression in response to acidosis, and TDAG8, whose acidification-induced action in osteoblasts is still under debate [90]. Conversely, the involvement of TDAG8 in counteracting the enhanced bone resorption typically associated with osteoporosis is well established [81].

In conclusion, several types of ASICs, TRPV ion channels, and GPCRs are involved in both osteoblast and osteoclast proton-sensing mechanisms. Their activation is likely to induce specific signaling cascades that, in turn, modulate the survival, differentiation, and activity of bone cells. A better understanding of the molecular mechanisms underlying the functioning of proton-sensing receptors and ion channels in the normal cells of the BM microenvironment will help to clarify if and how they can be targeted to treat patients with BM, in addition to add insights into the normal bone pathophysiology.

## Effects of acidosis on bone cells of the BM microenvironment

It is now widely accepted that interstitial acidification is a hallmark of solid tumor tissues since it contributes to tumor pathogenesis and progression by directly enhancing local invasiveness and aggressiveness, or by modulating therapy sensitivity, inhibiting immune surveillance, and drug resistance of cancer cells [11]. Like for other metastatic sites, tumor-generated acidosis in BM favors local invasiveness. However, in this case, local invasiveness might be enhanced also through the effect of tumor-derived extracellular acidosis on normal cells of the TME. Indeed, bone cells are extremely sensitive to the direct effects of pH. pH is so important for bone biology that the skeleton contains a large reserve of alkaline mineral (hydroxyapatite) [96] or buffer systems, like citrate [97], which is promptly available to neutralize metabolic H^+^ if the acid-base balance is not maintained within narrow limits. However, in pathological conditions, like cancer, the maintenance of this acid-base balance is altered by the proton extrusion of highly glycolytic cancer cells, and in the tumor osteolytic lesions, also by the acidification of tumor-induced hyperactivated bone-resorbing osteoclasts. It is interesting to note that osteoporotic bone, that is notoriously associated with interstitial acidosis [97], has been argued to be fertile soil for the development of BM [98, 99].

Most data on the effects of lowering the pH on bone cells come from studies not related to cancer: systemic acidosis has detrimental effects on the skeleton, and local acidosis coincides with bone destruction in inflammatory disease.

### The osteoclasts

Reduction of local pH stimulates the differentiation of osteoclast precursors into mature osteoclasts in the late phase of osteoclastogenesis, just before large-scale cell fusion [97, 100, 101]. Acidification also dramatically enhances the activity of osteoclasts and bone resorption, ultimately leading to bone loss [102]. Low pH stimulates bone erosion by activating mature osteoclasts already present in the bone rather than increasing the number of osteoclasts [102–104]. Other authors also demonstrated that an excess of extracellular H^+^ leads to decrements in intracellular pH and calcium, and promotes cell-matrix attachments by stimulating the expression of cell-matrix proteins that form podosomes [103, 105]. Acidification of osteoclasts also rapidly increases expression of CAII [106] and the V-ATPase [107], and strongly upregulates the osteoclast-specific collagenase cathepsin K. Furthermore, lowering extracellular pH dramatically increases the accumulation of the osteoclastogenic transcription factor, nuclear factor of activated T cells, cytoplasmic 1 (NFATc1) in nuclei of rat and rabbit osteoclasts to levels comparable with those induced by the pro-resorptive cytokine RANKL [78]. However, unlike RANKL, acidosis does not appear to induce the translocation of NF-κB to the nucleus. RANKL-stimulated NFATc1 nuclear translocation is the strongest and the main inducer of bone resorption in mature osteoclasts [33], both through the direct promotion of osteoclast differentiation and through the enhancement of the osteolytic activity. Thus, RANKL is a crucial factor for osteoclast differentiation, whereas both low pH and RANKL stimulus can equally promote and enhance osteoclast activity.

Finally, the effects of extracellular pH on lifespan are rather less clear-cut than the effects on differentiation and activation of osteoclasts although very few reports showed that acidosis suppresses the induction of apoptosis [108].

### The osteoblasts

**Fig. 3:**
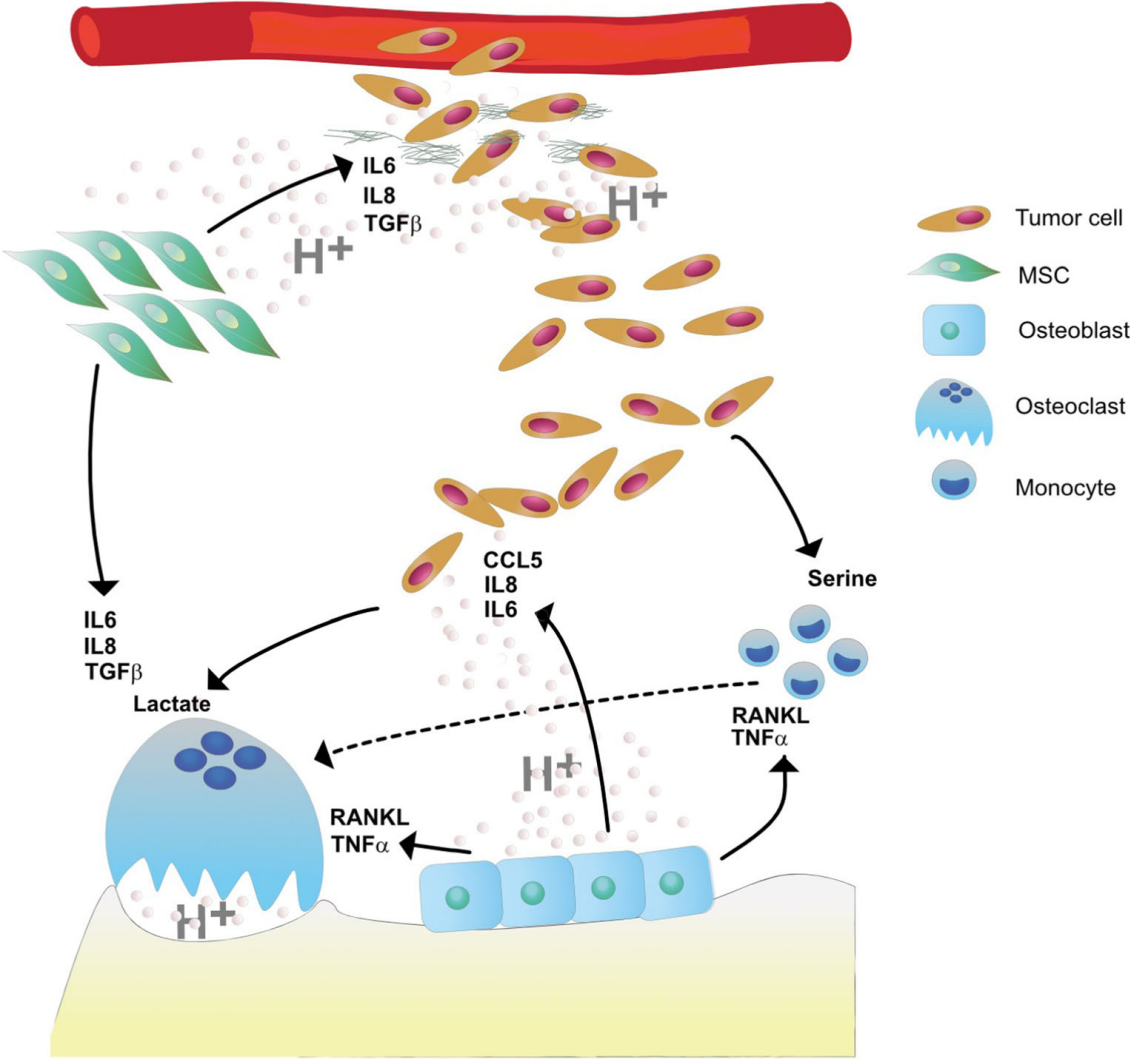
Acidosis-mediated interactions between the cells of BM microenvironment. Metastatic tumor cells colonize the bone through extravasation. In the bone microenvironment, these highly aggressive tumor cells release huge amount of protons, metabolites, and protein factors, which contribute to form the BM microenvironment. In particular, serine released by aggressive cancer cells promotes osteoclastogenesis from monocytic precursors, whereas lactate produced by glycolytic tumor cells is uptaken by mature osteoclasts to be metabolically recycled. Extracellular acidosis derived both from cancer cells and tumor-stimulated osteoclasts reprograms osteoblasts and MSC into an osteoclast-supporting phenotype by inducing an increased secretion of RANKL and TNF-α, and of IL6, IL8, and TGF-β, respectively. These cytokines, in turn, promote both the differentiation of monocyte precursors and the osteoclast-mediated bone resorption activity ultimately leading to BM-associated osteolytic lesions. Tumor acidosis may also educate stromal cells of the bone microenvironment toward a tumor-supporting phenotype by inducing the release of the pro-tumorigenic cytokines IL6, IL8, and CCL5 by osteoblasts, and IL6, IL8, and TGF-β by MSC

Extracellular acidosis inhibits most of the biological functions of osteoblasts, by impairing the activity of alkaline phosphatase, an enzyme crucial for bone mineralization, by affecting osteoblast differentiation and the osteoblast production of extracellular matrix that, in the end, results into an impaired formation of trabecular bone [96]. A low pH may also reprogram osteoblasts into an osteoclast-supporting phenotype. Indeed, extracellular acidosis induces an increased expression of the pro-osteoclastogenic RANKL in osteoblasts, both at mRNA and protein level, without affecting M-CSF and OPG expression [109, 110] (Fig. 3). This effect is possibly mediated by the acidosis-induced activation of OGR1/G(q/11)/phospholipase C/protein kinase C pathway with downstream cyclooxygenase 2 (COX-2) stimulation, and subsequent production of prostaglandin E2 (PGE2), a well-established inducer of RANKL expression [77]. Okito et al. have also shown that RANKL is activated *via* the adenylyl cyclase (AC)/cyclic adenosine monophosphate (cAMP) signaling that is induced by the proton receptor GPR4 [80]. Furthermore, acidosis reprograms osteoblasts to express other regulators of osteoclast biology, such as TNF-α [110] and the PTH/PTHrP receptors [111]. Finally, acidosis may educate osteoblasts toward a tumor-supporting phenotype. We have recently demonstrated that short-term exposure of osteoblasts to pH 6.8 promotes the expression of factors that foster tumor progression, such as interleukin 6 (IL6), interleukin 8 (IL8), and the chemokine (C-C motif) ligand 5 (CCL5) [30].

Overall, data from literature suggest that an acid microenvironment in BM simultaneously impairs bone formation and promotes in osteoblasts a pro-osteoclastogenic phenotype and a cancer-supporting phenotype, thereby exacerbating bone destruction and tumor growth.

### Mesenchymal stromal cells and cancer-associated fibroblasts

As for other cancers, BM behave as tissues or wounds that never heal [112], and MSC are closely involved in such a mechanism. MSC are osteoblast precursors that reside in the bone marrow and are also metabolically active cells that, when stressed by external stimuli, secrete a number of factors with paracrine activity. Notably, MSC have a strong tropism for tumors, engraft with TME, and respond to inflammatory mediators as deputies in the shelter of tissue. Although the distinction between MSC and cancer-associated fibroblasts (CAF) is still a matter of debate, it is believed that, in the TME, MSC can transdifferentiate into CAF, thereby fostering tumor progression [113, 114]. It is a matter of fact that CAF share several features with tumor-associated MSC, including the expression FAP, ACTA2, VEGF-AA, and IL6 [115]. However, the actual role of MSC in cancer is still controversial since either stimulating or inhibiting effects on tumor progression have been reported.

Under physiological conditions, lowering of pH (pH decrease of at least 0.5–1.0 pH units) is a driving force for the regenerative processes in bone. In a rat skeletal repair model, during the early healing phase, the pH showed to be lower than normal serum pH, whereas it increased to more alkaline values during the subsequent mineral deposition phase, thereby suggesting that the pH of repair tissue fluids plays a regulatory role in the healing and mineralization of bone [116]. In this context, in addition to osteoblasts and osteoclasts, MSC might be strongly implicated in the effect of pH on bone healing, mainly because of their reactive nature and inflammatory regulation abilities, especially during the early healing phase. According to our data, extracellular acidosis endows the maintenance of stemness in MSC by inducing the expression of stemness-related genes and proteins, and by driving MSC to reside in the quiescent G0 alert [117]. In addition, accumulating evidence has shown that low pH prompts MSC to express IL8 [118] and TGF-β [119]. In particular, TGF-β is involved in further recruitment of MSC at the bone site for their differentiation for subsequent bone formation [120]. These two factors are also well-recognized players in the vicious cycle of bone metastasis [121, 122]. Exploring this further, we have found that, like for hypoxia [123], extracellular acidosis in MSC activates the NF-κB inflammatory family of transcription factors *via* induction of RelA, RelB, and p50 expression, and the nuclear internalization of the NF-κB complex [124]. Acid-induced NF-κB activation downstream promotes the secretion of several cytokines, chemokines, and growth factors (IL1a, IL1b, IL6, IL8, IL23a, CCL5, CCL7, CXCL2, GM-CSF, and G-CSF) [125] that can elicit local cancer aggressiveness, tumor immune escape, and tumor-induced nociception and hyperalgesia. Finally, in the context of BM, inflammatory factors that are released by acid-induced MSC may also indirectly prompt the osteoclast differentiation and activity, like IL6 which promotes bone resorption *via* the same mechanism as PTHrP, i.e., by stimulating RANKL expression and inhibiting the expression of its decoy receptor, OPG, by osteoblasts [4, 126].

## Cancer-induced bone pain

**Fig. 4:**
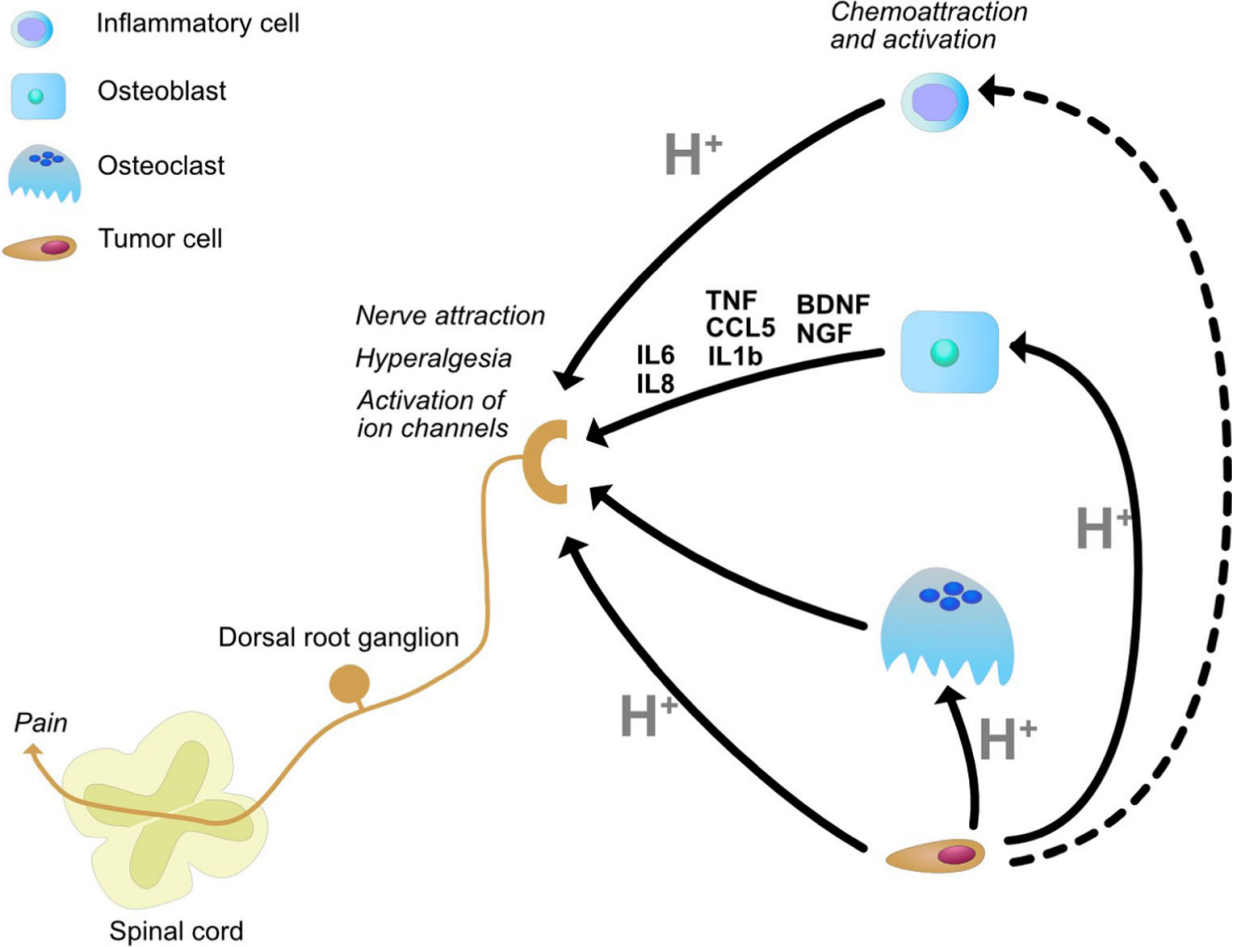
Mechanisms of acid-induced CIBP in BM microenvironment. The extracellular acidosis derived from cancer cells, tumor-induced osteoclasts, and inflammatory cells in the BM microenvironment directly activate acid-sensing ion channels on the membrane of the nociceptor terminals on sensitive neurons in bone. This nociceptor rapidly converts the noxious stimuli into electrochemical signals and transmits them to brain through dorsal root ganglion (primary afferent neuron) and spinal cord (secondary afferent neuron) to induce pain. In addition to its direct algogenic effect, acidosis also prompts the release of inflammatory and nociceptive mediators (IL6, IL8, TNF, CCL5, IL1b, BDNF, NGF) from the tumor-associated osteoblasts that further enhance CIBP by promoting nerve attraction, hyperalgesia, and nociceptor sensitization

Pain is a common event in patients with BM, [127] resulting in anxiety and depression, reduced performance status, and impairment of life quality [128]. Although still incompletely unveiled, the biological mechanisms underlying cancer-induced bone pain (CIBP) involve a complex interplay among the tumor cells, peripheral nerves, and cells of the bone. CIBP has to be conceived as a mixed type of chronic pain that includes both neuropathic and inflammatory nociceptive pain. The ability of tumor growth to injure, distend, and entrap the primary afferent fibers leads to neuropathic pain [2]. However, the sensory neurons (nociceptors) densely innervate both the trabecular bone and the periosteal surfaces of the cortical bone [129, 130] and may also lead to neuropathic pain. Thus, the release of extracellular protons in BM microenvironment by cancer cells, tumor-induced osteoclasts, and inflammatory cells may directly stimulate the acid-sensing ion channels that are expressed on sensory neurons, thereby evoking an algogenic signal [131]. Acid-induced bone pain has been mainly related to the acid-sensing ion channels TRPV1 and ASIC3 [131]. It should be taken into account that a variety of nociceptive and inflammatory mediators and neuromodulators are also released by injured tissues or by bone, tumor, and inflammatory cells that can further activate and sensitize nociceptors [132] (Fig. 4). Indeed, we recently demonstrated both in *in vitro* and *in vivo* models that the low pH in the BM microenvironment exasperates the release of the same nociceptive and inflammatory mediators by bone cells [30]. Among them, the nerve growth factor (NGF) [133] and the brain-derived neurotrophic factor (BDNF) [134] cause axonal chemoattraction, and IL-6, IL-8, IL1b, and CCL5 foster tumor-associated hyperalgesia [30].

In conclusion, intratumoral acidification promotes CIBP in BM by a direct acid-induced stimulation of nociceptors in bone, and by activating the tumor-associated stroma that, in turn, fosters hyperalgesia through the release of an inflammatory secretome. The evidences of cancer-associated acidosis as an additional stimulus for bone pain adds new insights into the complex pathogenesis of CIBP that help to explain the lack of responsiveness to conventional anti-analgesic drugs in patients with BM and that may be used for the development of novel palliative treatments in advanced cancer.

## Targeting acidosis in bone metastasis

Targeting the mechanisms responsible for proton extrusion or proton detection may represent an appealing strategy for the development of new and less toxic treatment of BM. To address this, several *in vivo* models of BM have been developed [135], especially for the identification of novel pain killers [136].

Neutralization of tumor-derived acid with systemic buffer therapies, like sodium bicarbonate and lysine, was proposed several years ago with promising results in animal models of cancer that also included breast carcinoma cells that very often metastasize to the bone [137–139]. Furthermore, although buffer therapies have never been considered to specifically treat BM, clinical trials on human based on NaHCO_3,_ on a urease that can raise pH by converting endogenous urea to two NH_4_^+^, or on the hydrochloric acid binder TRC101 have been also proposed [140]. One major concern of buffer therapies is the lack of specificity, and thus the occurrence of possible systemic toxicity [140]. With a more specific approach, several novel V-ATPase inhibitors have been investigated for the same purpose, including bafilomycin A1 [141], but none of them has been yet translated to the clinic due to their unspecific and high toxic effects. In this context, omeprazole and related gastric H^+^/K^+^-ATPase inhibitors can also inhibit the V-ATPase by binding the subunit A of the nucleotide binding domain [142] and have recently sparked great interest showing significant results in reducing CIBP in a model of BM from breast carcinoma [30] (and Logazziano, this volume). Also for these kinds of drugs, very few clinical trials to avoid intratumoral acidosis in human and in companion animals with spontaneous cancer have been reported showing enhanced antitumor effects of chemotherapy [143–145]. Not least, these drugs are widely prescribed as pivotal treatment of peptic diseases, with minimal side effects even when administered at high doses and are thus very promising. An additional attractive feature of these agents is that they require acid conditions to be converted into the active form, therefore providing the possibility of BM-specific selection. The V-ATPase inhibitor bafilomycin A1 has been also used to decrease bone pain in mouse preclinical models of multiple myeloma [146] and of inflammatory pain [147], but most of the current literature about CIBP management has been focused on the effect of ASIC3 and TRPV1 inhibitors. One example is the use of the specific ASIC3 antagonist APETx2 [148, 149], or the selective blockage of TRPV1 by the specific inhibitor JNJ-17203212 in a mouse model of osteolytic sarcoma, another type of bone cancer [150]. Other drugs targeting TRPV1 that have been successfully used in *in vivo* preclinical models are QX-314 [151] and SB366791 [152].

CAs are other attractive therapeutic targets, especially in the context of BM, since CAII is expressed by osteoclasts, whereas CAIX is highly expressed by carcinoma cells and their inhibition would simultaneously affect different aggressive mechanisms involved in BM progression. Chemical inhibitors against CAs have been tested in several clinical trials in humans (more than 100 results in clinicaltrials.gov by searching for the solely CA inhibitor “acetazolamide”). Among these, drugs against CAIX have been used in preclinical *in vivo* models revealing a great potential in inhibiting the formation of metastases in several types of cancer [153, 154] and have been also used in cancer patients [155], like for the CAIX inhibitor SLC-0111 (ClinicalTrials.gov Identifier: NCT02215850 Phase I, NCT03450018 Phase Ib). However, they have never been considered to specifically treat BM patients. It is noteworthy to mention the attempt by Tauro et al. that developed dual CA/matrix metalloproteinase inhibitors incorporating bisphosphonic acid [156]. This compound has been developed to specifically target acidifying tumors that invade bone, since it targets both the collagenase MMP and the CA, and bisphosphonate, a bone-targeting molecule that may carry the two anticancer drugs directly to the site of tumor-induced osteolysis.

## Conclusions

Although, to date, different methods have been developed to measure the pH in cancer [157], the real interstitial pH in bone metastasis has never been quantified, mostly due to technical difficulties. However, it is very likely that BM has quite an acid TME that has unique features, with a major impact on both local tumor aggressiveness and bone tissue homeostasis, and on the induction of cancer-induced bone pain. We discussed and summarized the most recent literature in the field showing that in BM microenvironment, (1) the extracellular space is largely protonated both by the highly glycolytic tumor cells and the highly acidifying osteoclasts degrading bone, and that (2) bone cells and nociceptors that innervate bone are very sensitive to extracellular acidification. The high extracellular concentration of hydrogen ion in BM microenvironment is thus responsible for altering bone homeostasis, enhancing local tumor aggressiveness and osteolysis, and promoting local inflammation and cancer-induced bone pain.

In conclusion, intratumoral acidosis offers as a promising target for novel and more effective anticancer and palliative treatments for patients with advanced cancer that urges to be more deeply considered and explored.

## Abbreviations

BM: Bone metastasis
MSC: Mesenchymal stromal cells
TME: Tumor microenvironment
ECM: Extracellular matrix
V-ATPase: Vacuolar ATPase
NHE: Na+/H+ exchanger
MCT: Monocarboxylate transporters
CAIX: Carbonic anhydrase 9
CAII: Carbonic anhydrase 2
M-CSF: Macrophage colony-stimulating factor
NF-κB: Nuclear factor-kappa B
RANKL: Nuclear factor-kappa B ligand
CIC-7: Chloride channel 7
NBCn1: Na^+^-HCO3^−^ co-transporter
AC: Adenylyl cyclase
ACTA2: Alpha smooth muscle actin 2
AE2: Cl^−^/HCO3^−^ anion exchanger
ASIC: Acid-sensing ion channels
cAMP: Cyclic adenosine monophosphate
CCL: C-C motif ligand
CIBP: Cancer-induced bone pain
CXCL: C-X-C motif ligand
FAP: Fibroblast-activating protein
GAPDH: Glyceraldehyde 3-phosphate dehydrogenase
GM-CSF: Granulocyte-macrophage colony-stimulating factor
GPCR: Proton-sensing G protein-coupled receptors
GPR: G protein-coupled receptor
IL: Interleukin
NFATc1: Nuclear factor of activated T cells, cytoplasmic 1
NHERF1: Na^+^/H^+^ exchanger regulatory factor
OGR1: Ovarian cancer G protein-coupled receptor 1
PKM2: Pyruvate kinase muscle isozyme 2
PTHrP: Parathyroid hormone-related protein
TGF-β: Transforming growth factor beta
TNF-α: Tumor necrosis factor alpha
TRAF-6: TNF receptor-associated factor 6
TRAP: Tartrate-resistant acid phosphatase
TRPV: Transient receptor potential vanilloid
VEGF-AA: Vascular endothelial growth factor A-A precursor
